# Association of sarcopenia with treatment tolerance in older adults with acute myeloid leukemia using artificial intelligence computed tomography–segmentation and a geriatric assessment

**DOI:** 10.1038/s41375-026-03099-7

**Published:** 2026-08-31

**Authors:** Samuel J. Yates, Elizabeth Terman, Lauren Miller, John F. Cursio, Claire Wild, Megan Huisingh-Scheetz, Michael Zhang, Nicholas Feinberg, Karteek Popuri, Mirza Faisal Beg, Varun Akella, Hamed Youshanlouei, Afsheen Noorani, Jamie Mathews, Caner Saygin, Anand A. Patel, Olatoyosi Odenike, Richard A. Larson, Mariam T. Nawas, Wendy Stock

**Affiliations:** 1 Section of Hematology and Oncology, University of Chicago, Chicago, IL USA; 2 Department of Internal Medicine, University of Chicago, Chicago, IL USA; 3 Department of Therapy Services, University of Chicago, Chicago, IL USA; 4 Department of Public Health Sciences, University of Chicago, Chicago, IL USA; 5 Pritzker School of Medicine, University of Chicago, Chicago, IL USA; 6 Section of Geriatrics, University of Chicago, Chicago, IL USA; 7 Department of Radiology, University of Chicago, Chicago, IL USA; 8 Department of Computer Science, Memorial University of Newfoundland, St. John’s, NL Canada; 9 School of Engineering Science, Simon Fraser University, Vancouver, BC Canada

**Keywords:** Clinical trials, Acute myeloid leukaemia

## Abstract

**Figure:**
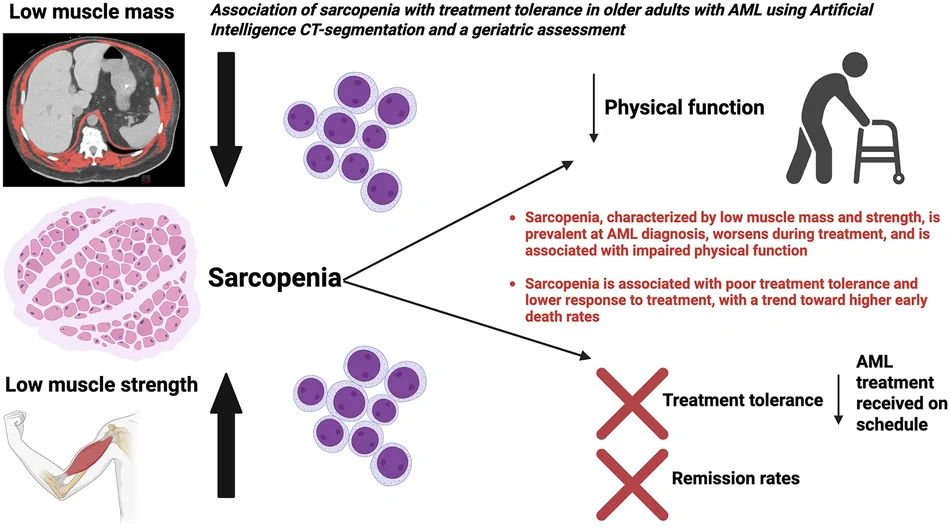

## Introduction

Less intensive treatment (LIT) regimens, primarily the combination of a hypomethylating agent and venetoclax (HMA+venetoclax), have replaced intensive regimens for the frontline treatment of AML for most older adults [1]. However, poor treatment tolerance remains an issue for older adults receiving LIT. Older adults receiving HMA+venetoclax continue to experience high 60- and 90-day mortality (10–20%), with frequent dose reductions and treatment discontinuations [2]. Patients value preservation of physical function as a measure of treatment tolerance [3]. Impaired physical function also predicts poor treatment tolerance and shorter survival in older adults receiving intensive and LIT regimens [4]. Evidence-based strategies to improve treatment tolerance represent a care gap in the management of older adults with AML.

Sarcopenia, defined as low muscle mass (myopenia) and strength (dynapenia), is a driver of poor treatment tolerance in older adults with solid tumors, but studies in AML are lacking [5]. Sarcopenia is established in geriatrics as prevalent (5–13% of adults aged 60–70 years) and clinically significant; multiple studies demonstrate associations with impaired physical function, reduced functional status, and increased all-cause mortality [5]. Further, interventions to mitigate sarcopenia are effective [6]. Previous studies of sarcopenia in patients with AML have only assessed myopenia, included few older adults, and primarily included patients receiving intensive regimens [7]. To address this knowledge gap, we conducted a prospective observational study of older adults with newly diagnosed AML to assess the prevalence of sarcopenia, changes in muscle mass and strength during AML treatment, and the impact of sarcopenia on treatment tolerance. We hypothesized that subjects with sarcopenia would have poorer treatment tolerance than subjects without sarcopenia.

## Methods

This study (ClinicalTrials.gov: NCT05458258) was conducted at the University of Chicago Comprehensive Cancer Center (UCCCC). The UCCCC Institutional Review Board approved the study. All subjects provided informed consent. Inclusion criteria were as follows: newly diagnosed AML, age ≥ 60 years, and plan to receive AML-directed treatment. Sarcopenia and a geriatric assessment (GA) were collected at two time points: prior to AML treatment initiation and after one cycle of AML treatment. Sarcopenia was defined by the combination of myopenia from computed tomography-derived skeletal muscle index (SMI) and dynapenia by grip strength [5]. Skeletal muscle was quantified as area (cm^2^) localized to the twelfth thoracic vertebrae (T12) level. T12 selection, image segmentation, and tissue area quantification were conducted via an automated deep learning-based pipeline (DAFS-CT platform, FDA 510K-cleared, Voronoi Health Analytics Inc., Vancouver, British Columbia, Canada) [8, 9]. We used a GA aligned with recommendations from the American Society of Hematology and American Society of Clinical Oncology [10, 11]. Measures and thresholds for impairment are found in Table S1. Disease risk was assigned according to the 2024 European LeukemiaNet (ELN) less-intensive risk classification [12].

Response to AML treatment (complete response (CR) and complete response with incomplete count recovery (CRi)), survival, and early death (60- and 90-day mortality) were assessed using the 2022 ELN AML response criteria [13]. Treatment tolerance outcomes were early death and the ability to complete two cycles of HMA+venetoclax within 90 days of treatment start [4]. The primary endpoint was the association between sarcopenia and 60-day mortality. A sample size of 82 patients provided 80% power to detect a hazard ratio of 3.0 for 60-day mortality using a Cox proportional hazards model, assuming a 60-day mortality rate of 18%. Optimum stratification was used to derive sex-specific SMI thresholds that best discriminated risk of the time-to-event outcome (death) [14]. These cutoffs were then used to classify patients as having myopenia (low SMI) or not. The multivariable model assessing the relevance of sarcopenia on the primary outcome was prespecified to adjust for age, disease risk, performance status, treatment intensity, and GA variables predictive of treatment tolerance. No adjustment for multiple comparisons was performed; therefore, analyses other than the primary outcome analysis should be considered exploratory. All analyses used SAS version 9.4 (SAS Institute, Cary, North Carolina, USA).

## Results

The target 82 participants were enrolled between 1 March 2023 and 18 April 2025 (Fig. S1). Seventy-eight subjects were included in the final analysis. Median age of the cohort was 73 years (range, 60–93) (Table S2). Most subjects (64/78) received LIT regimens; 53/78 of the cohort received HMA+venetoclax. Seven subjects died before initiating AML-directed therapy. The prevalence of myopenia, dynapenia, and sarcopenia was 46/78, 36/72, and 22/74, respectively. Malnutrition (58/73) and cachexia (28/78) were common. Impairments in physical function were frequent, as demonstrated by deficits in the 6-minute walk test (6MWT) (50/67) and timed up and go (TUG) test (46/74). Missing data were minimal (Table S3).

Median survival for the whole cohort was 8 months (range, 4 days-28 months); survival was 8.6 months when excluding those who died before initiating AML treatment. 60- and 90-day mortality occurred in 20/78 and 24/78, respectively, for the whole cohort and 13/71 and 17/71, respectively, when excluding those who died before initiating AML treatment (Table S4). CR/CRi to first-line treatment was achieved in 38/78 of the cohort and 38/71 when excluding those deceased before undergoing the end of C1 response assessment. Among subjects who received an HMA+venetoclax-based regimen, 40/60 were able to complete two cycles within 90 days of treatment initiation. Of the 14 subjects who received an HMA+venetoclax-based regimen and died within 90 days, only two were able to receive two cycles, but 13/14 had a response of morphologic leukemic-free state, partial response, or stable disease. Causes of death for 10/14 of these subjects were unrelated to progression of disease (Table S5).

**Fig. 1:**
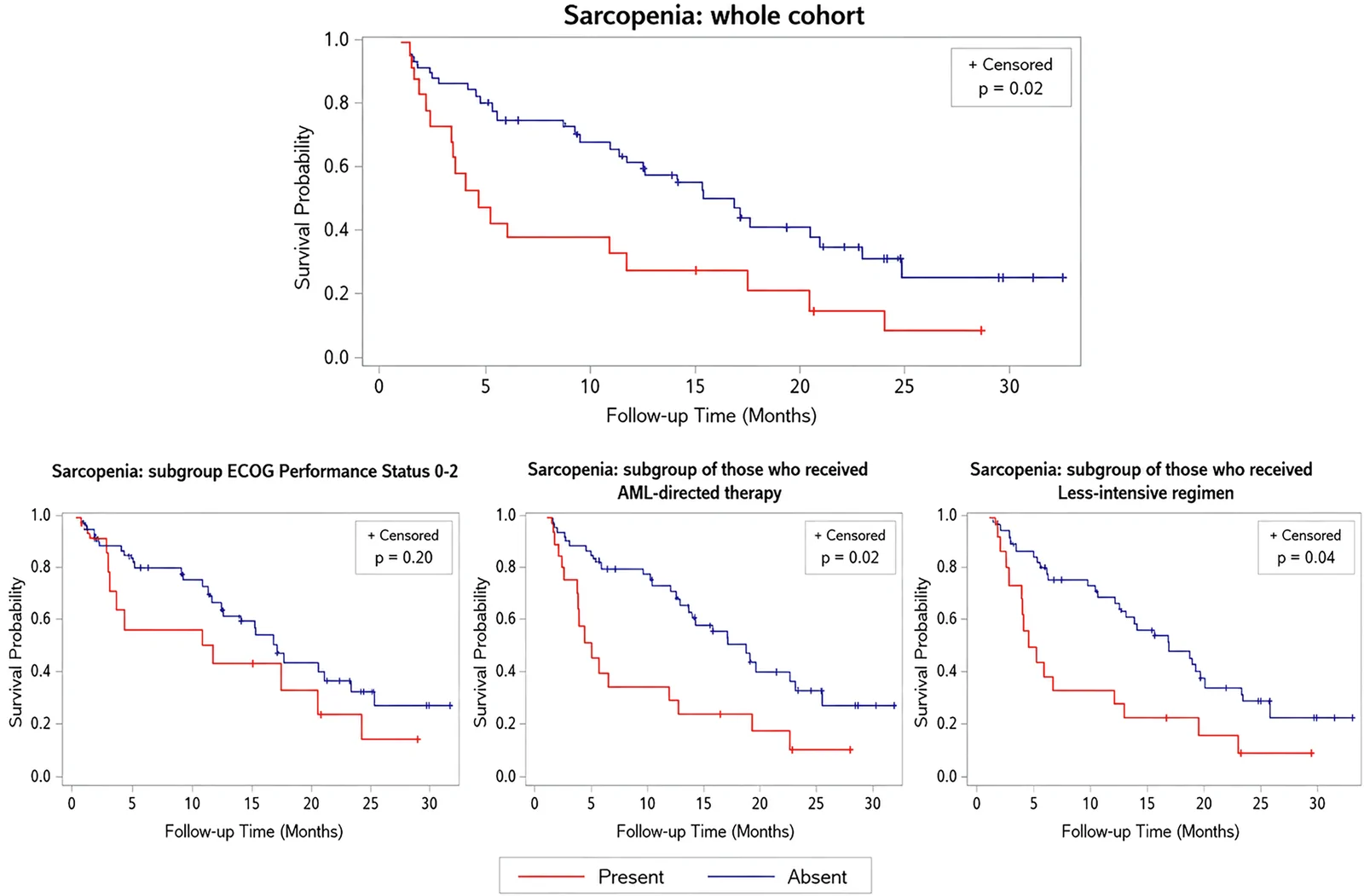
Overall survival stratified by presence of sarcopenia at diagnosis of AML. Those with sarcopenia had shorter survival in the whole cohort and pre-specified subgroups except for the subgroup of those fit by ECOG performance status. The sample size for each curve is: whole cohort (*n* = 74), ECOG Performance Status 0–2 (*n* = 59), received AML-directed therapy (*n* = 68), received Less-intensive treatment regimen (*n* = 61).

Subjects with sarcopenia had higher rates of 60- and 90-day mortality (Table S4) and shorter survival (Fig. 1). In multivariable analyses, sarcopenia was independently associated with lower odds of receiving 2 cycles of an HMA+venetoclax-based regimen within 90 days, lower odds of achieving a CR/CRi, and lower utilization of allogeneic stem cell transplant (alloSCT) in CR1, including in prespecified subgroups (Table 1 and Fig. S6). Due to concerns for model overfitting in the prespecified models, we conducted a sensitivity analysis using a stepwise model-building procedure (Table S7). Sarcopenia was independently associated with 90-day mortality, lower odds of receiving 2 cycles of an HMA+venetoclax-based regimen within 90 days, lower odds of achieving a CR/CRi, and lower utilization of allogeneic stem cell transplant (alloSCT) in CR1 in the sensitivity analysis. Among those who did not experience 90-day mortality, subjects with sarcopenia had numerically lower rates of receiving two cycles of HMA+venetoclax-based regimens (3/8 versus 4/37; *p* = 0.09) and lower CR/CRi rates compared to subjects without sarcopenia (5/10 versus 33/43; *p* = 0.12). Myopenia was independently associated with all treatment tolerance outcomes and lower odds of achieving a CR/CRI (Table 1 and Fig. S6). We also conducted a sensitivity analysis using muscle mass continuously (raw SMI data) (Table S8). SMI retained significance for outcomes aside from 60-day and 90-day mortality. In multivariable analyses, those with an impaired TUG test had higher 60- and 90-day mortality, lower odds of receiving alloSCT in CR1, and shorter survival (Table 1 and Fig. S6 and Fig. S2). Results were similar when evaluating treatment tolerance by impairment on the 6MWT.

**Table 1:** Univariate and multivariable models evaluating sarcopenia, myopenia, dynapenia, and physical function and their impact on treatment outcomes.

Outcome	60-day mortality: Hazard Ratio [95% CI], *p*-value, c-statistic	90-day mortality: Hazard Ratio, [95% CI], *p*-value, c-statistic	Ability to finish 2 cycles of HMA/Ven: Odds Ratio [95% CI], *p*-value, c-statistic	CR/CRi:Odds Ratio [95% CI], *p*-value, c-statistic	Receipt of AlloSCT: Odds Ratio [95% CI], *p*-value, c-statistic	Overall survival: Hazard Ratio [95% CI], *p*-value, c-statistic
**Sarcopenia**						
Univariate	3.50 [1.30, 9.43], ***p*** = **0.01**, c = 0.64	3.56 [1.47, 8.62], ***p*** = **0.01**, c = 0.64	0.19 [0.06, 0.64], ***p*** = **0.01**, c = 0.66	0.17 [0.05, 0.53], ***p*** = **0.01**, c = 0.67	**Inestimable, 0 events in sarcopenic group**	2.03 [1.13, 3.64], ***p*** = **0.02**, c = 0.59
Multivariable	1.96 [0.52, 7.42], *p* = 0.32, c = 0.84	2.53 [0.79, 8.13], *p* = 0.12, c = 0.83	0.19 [0.04, 0.95], ***p*** = **0.04**, c = 0.82	0.24 [0.07, 0.89], ***p*** = **0.03**, c = 0.83	**Inestimable, 0 events in sarcopenic group**	1.63 [0.71, 3.73]. *p* = 0.21, c = 0.77
**Myopenia**						
Univariate	7.14 [1.65, 31.25], ***p*** = **0.01**, c = 0.68	4.20 [1.43, 12.35], ***p*** = **0.01**, c = 0.65	0.24 [0.08, 0.71] ***p*** = **0.01**, c = 0.67	0.24 [0.09, 0.64], ***p*** = **0.01**, c = 0.66	0.27 [0.08, 0.88], ***p*** = **0.03**, c = 0.66	2.00 [1.13, 3.53], ***p*** = **0.02**, c = 0.61
Multivariable	5.56 [1.01, 30.48], ***p*** = **0.04**, c = 0.86	3.99 [1.12, 14.29], ***p*** = **0.03**, c = 0.84	0.20 [0.04, 0.95], ***p*** = **0.04**, c = 0.83	0.25 [0.07, 0.92], ***p*** = **0.04**, c = 0.90	0.30 [0.07, 1.42], *p* = 0.13, c = 0.92	1.57 [0.82,3.02], *p* = 0.18, c-0.74, c = 0.83
**Dynapenia**						
Univariate	6.37 [1.41, 28.57], ***p*** = **0.02**, c = 0.70	5.65 [1.62, 19.61], ***p*** = **0.01**, c = 0.69	0.29 [0.09, 0.90], ***p*** = **0.03**, c = 0.65	0.29 [0.11, 0.78], ***p*** = **0.01**, c = 0.65	0.10 [0.02, 0.48], ***p*** = **0.01**, c = 0.74	1.62 [0.90, 2.89], *p* = 0.11, c = 0.60
Multivariable	3.14 [0.59, 16.67], *p* = 0.18, c = 0.88	2.00 [0.51, 7.87], *p* = 0.32, c = 0.70	0.82 [0.20, 3.32], *p* = 0.78, c = 0.79	0.57 [0.14, 2.26], *p* = 0.42, c = 0.86	0.176 [0.02, 1.41], *p* = 0.10, c = 0.91	0.94 [0.42, 2.12], *p* = 0.89, c = 0.72
**TUG**						
Univariate	**Inestimable, 0 deaths in unimpaired group**	17.31 [2.33,28.9], ***p*** = **0.01**, c = 0.71	0.063 [0.01, 0.31], ***p*** < **0.001**, c = 0.75	0.28 [0.10, 0.75], ***p*** = **0.01**, c = 0.65	0.05 [0.01,0.26], ***p*** < **0.001**, c = 0.81	2.65 [1.44, 4.87], ***p*** = **0.01**, c = 0.65
Multivariable	**Inestimable, 0 deaths in unimpaired group**	15.26 [1.69, 138.12], ***p*** = **0.02**, c = 0.91	0.18 [0.03, 1.10], *p* = 0.06, c = 0.88	0.86 [0.18, 4.08], *p* = 0.85, c = 0.88	0.09 [0.01, 0.74], ***p*** = **0.02**, c = 0.95	2.28 [1.05, 4.95]. ***p*** = **0.04**, c = 0.83
**6MWT**						
Univariate	**Inestimable, 0 deaths for unimpaired group**	**Inestimable, 0 deaths for unimpaired group**	0.09 [0.01, 0.73], ***p*** = **0.02**, c = 0.65	0.14 [0.4, 0.54], ***p*** = **0.01**, c = 0.66	0.03 [0.01, 0.14], ***p*** < **0.0001**, c = 0.85	2.61 [1.21, 5.61], ***p*** = **0.01**, c = 0.61
Whole cohort	**Inestimable, 0 deaths for unimpaired group**	**Inestimable, 0 deaths for unimpaired group**	0.26 [0.02, 3.33], *p* = 0.30, c = 0.88	0.26 [0.031, 2.22], *p* = 0.22, c = 0.91	0.002 [ < 0.001, 0.37], ***p*** = **0.02**, c = 0.99	1.73 [0.63, 4.78], *p* = 0.29, c = 0.83
**SPPB**						
Univariate	13.82 [1.84, 103.86], ***p*** = **0.01**, c = 0.70	5.36 [1.58, 18.13], ***p*** = **0.01**, c = 0.67	0.17 [0.05, 0.59], ***p*** = **0.01**, c = 0.68	0.23 [0.09, 0.61], ***p*** = **0.01**, c = 0.67	0.06 [0.01, 0.29], ***p*** < **0.001**, c = 0.79	2.66 [1.45, 4.88], ***p*** = **0.01**, c = 0.63
Whole cohort	5.29 [0.60, 46.71], *p* = 0.13, c = 0.92	2.02 [0.51, 8.00], *p* = 0.32, c = 0.88	0.57 [0.13, 2.43], *p* = 0.44, c = 0.85	0.83 [0.21, 3.29], *p* = 0.79, c = 0.84	0.21 [0.03, 1.43], *p* = 0.11, c = 0.93	1.57 [0.75, 3.28]. *p* = 0.23, c = 0.82

The multivariable model for the impact of sarcopenia on 60-day mortality was prespecified to adjust for age, ELN 2024 risk category, ECOG PS, treatment intensity (intensive or less-intensive regimen), and GA variables with prior literature supporting their impact on treatment tolerance (MOCA, CCI, IADL, and SPPB). We also applied these models to the other treatment-tolerance outcomes: 90-day mortality and the ability to receive 2 cycles of HMA+venetoclax within 90 days. Similarly, we applied these covariates to myopenia and dynapenia given they are components of sarcopenia. The event-per-variable (EPV) ratios were: 60-day mortality (2.2), 90-day mortality (2.7), and ability to receive 2 cycles of HMA+venetoclax within 90 days (4.4). Final models for the impact of sarcopenia, myopenia, and dynapenia on response, receipt of alloSCT, and OS and all models for physical function tests included: age, performance status, European LeukemiaNet 2024 less-intensive risk classification, and treatment intensity. Additional covariates, including CCI, MOCA, IADL, and SPPB, were tested but not retained in the final model due to instability. EPV ratios were: CR/CRi (7.6), receipt of alloSCT (3.0), death (11.0). C statistic of 0.5 indicates the model predicts outcome as well as chance; 0.7 to 0.8 indicates acceptable discrimination; 0.8 to 0.9 indicates excellent discrimination; 0.9 to 0.99 is outstanding discrimination; 1.0 is perfect prediction. *AML* acute myeloid leukemia, *alloSCT* allogeneic stem cell transplantation, *CR* complete remission, *CRi* complete remission with incomplete hematologic recovery, *ECOG PS* Eastern Cooperative Oncology Group performance status, *HMA/Ven* hypomethylating agent plus venetoclax, *SD* standard deviation, *TUG* Timed Up and Go, *6MWT* 6-minute walk test, *SPPB* Short Physical Performance Battery.

No differences in rates of individual mutations and disease risk scores were observed by sarcopenia (Tables S9–11). Myopenia showed statistically significant, weak correlations with severity of malnutrition, cachexia, and impairment in physical function tests (Table S12). Dynapenia showed statistically significant, strong correlations with malnutrition and physical function testing.

43 subjects completed some or all repeat sarcopenia and GA measures. Early death was the most common reason for missing data (Table S3). Most subjects experienced losses in muscle mass (29/43, median change in SMI = −1.93 cm^2^/m^2^) and muscle strength (25/37; median change in grip strength = −1.0 kg) (Table S13). Median weight change was −1.6 kg. Half of the cohort had worse performance on the TUG test (median change of +0.3 s).

## Discussion

Utilizing a novel AI-imaging platform and GA, our study demonstrates that sarcopenia is common at AML diagnosis, worsens during early AML treatment, and is independently associated with some measures of treatment tolerance, lower rates of remission, and lower receipt of alloSCT among older adults. Notably, our study did not meet its primary treatment-tolerance endpoint: death within 60 days. However, the observed effect size was moderate and approached statistical significance. While our study was not powered to detect differences in overall survival by sarcopenia status, the observed effect size remained moderate, although not statistically significant, in the multivariable analyses. Myopenia, a component of sarcopenia, was independently associated with all outcomes related to treatment tolerance, including early death, and remission rates. We demonstrate that malnutrition and cancer cachexia correlate with the severity of sarcopenia, adding potential mechanistic insights into the development of sarcopenia among older adults with AML. Finally, we showed that sarcopenia is associated with impaired physical function, which in turn was independently associated with worse treatment tolerance and decreased survival, including early deaths. Collectively, our findings suggest that sarcopenia is associated with poor treatment tolerance in older adults with AML, potentially through its contribution to impaired physical function

Our findings have several implications for the field of AML and blood cancers in general. Despite having an ICD-10-CM diagnosis code (M62.84) assigned to sarcopenia in 2016, this condition is critically underrecognized. A recent study evaluating discordance between ICD-10 coding for sarcopenia and CT-defined myopenia in ~18,000 patients found substantial undercoding: 28.5% had myopenia, yet the ICD code was used in only 0.05% [15]. Second, our observational data support the rationale for interventional trials targeting sarcopenia. The mainstay of sarcopenia treatment in older adults without cancer is a high-protein diet (1.0–1.5 g/kg body weight/day) and resistance exercise training as established by the SPRINTT trial [6]. To date, no trials have translated this approach to older adults with blood cancers. Finally, our findings suggest that myopenia may provide greater prognostic information than the consensus definition of sarcopenia in older adults with AML. This hypothesis warrants further investigation in larger observational studies and pilot interventional trials. In particular, future studies should evaluate whether improvements in muscle mass alone are sufficient to enhance treatment tolerance, or whether concurrent improvements in both muscle mass and strength are necessary.

This study has several limitations. The cohort was derived from a single academic center and consisted predominantly of White subjects. Applicability of the results to more diverse populations and practice settings requires further study. Observed changes in muscle mass, muscle strength, and physical performance should be interpreted with caution due to potential survivor bias. The sex-specific T12 SMI thresholds were derived using outcome-based optimal stratification and subsequently evaluated within the same cohort, which created the potential for optimistic bias and overestimation of prognostic performance. However, sensitivity analyses modeling SMI as a continuous variable demonstrated generally consistent findings. The proposed cutoffs should be considered exploratory and require validation in independent cohorts with a larger sample size. The low event-per-variable ratios in our prespecified models raise the possibility of model overfitting. In a sensitivity analysis using a stepwise model-building approach that yielded higher event-per-variable ratios, the findings were similar, supporting the observed associations. However, the reported estimates from our models should be interpreted with appropriate caution. There are also limitations to using completion of two cycles of HMA+venetoclax within 90 days as a measure of treatment tolerance. This outcome may be influenced by disease progression, physician preference, and logistical factors. Though imperfect, this endpoint is relevant as it reflects the ability to maintain therapy during the period when treatment-related complications, dose interruptions, and early attrition are most common and prognostically relevant.

In conclusion, sarcopenia contributes to impaired physical function and is associated with poor treatment tolerance among older adults with AML. Interventional trials, including exercise and nutritional supplementation aimed at targeting sarcopenia and improving treatment tolerance, are warranted.

## Supplementary information

Final Supplement

## Data Availability

The datasets generated during and/or analysed during the current study are available from the corresponding author on reasonable request.
