# Supplementary material for: Association of sarcopenia with treatment tolerance in older adults with acute myeloid leukemia using artificial intelligence computed tomography–segmentation and a geriatric assessment
Source: Leukemia. 2026 Aug 31;40(11):2611–5. doi: 10.1038/s41375-026-03099-7 (PMC13713559; doi:10.1038/s41375-026-03099-7)
Supplement: Supplementary file 1 [file 41375_2026_3099_MOESM1_ESM.pdf]

Table S1. Sarcopenia and geriatric assessment results at diagnosis among 78 older adults with AML

GA Domain	Score defining impairment	No. (impaired/total)	% impaired	Median (Q1, Q3)
**Sarcopenia, cachexia, and nutrition**				
**Myopenia**<br><br>Impairment: low skeletal muscle mass	Females: SMI< 35.0 cm^2/m^2 for females at T12<br><br>Males: SMI< 36.0 cm^2/m^2 for males at T12	46/78	59%	31.1 (25.2, 37.9)<br><br>35.9 (30.0, 41.3)
**Dynapenia(1)**<br><br>Impairment: low grip strength on handgrip dynamometer	 <br>Females <16 kg<br><br>Males< 27kg	36/72<br><br>16/31<br><br>20/41	50%<br><br>Females: 53%<br><br>Males: 49%	 <br>Females: 15.3 (13.0, 20.5)<br><br>Males: 27.5 (22.0, 36.0)
**Sarcopenia**[*]	Myopenia + Dynapenia: Low SMI *and* low grip strength	22/74	30%	
**WLGS(2)**<br><br>Impairment: cancer cachexia	≥2	28/78	36%	1 (0,2)
**PG-SGA(3)**<br><br>Impairment: Malnutrition	≥4	58/73	79%	9 (4, 13)
**Albumin**	≤3.5 g/dL	37/78	47%	3.4 (2.9, 3.8)
**Pre-albumin**	≤20mg/dL	60/77	78%	14 (8.5, 19)
**Physical function**				
**6-MWT(4)**	<400 meters walked in 6 minutes	50/67	75%	169 (0, 389)
**SPPB(5)**	≤9	45/76	59%	8 (3,11)
**TUG(6)**	≤13.4 seconds	46/74	62%	13.6 (9.1, 21.3)
**Functional Status**				
**Katz ADL(7)**	<6	15/74	20%	6 (6, 6)
**Lawton IADL(8)**	<8	32/74	43%	8 (5, 8)
**Comorbidities**				
**CCI(9)**	≥1	47/78	60%	1 (0, 2)
**Cognition**				
**MOCA(10)**<br><br>Normal<br><br>Mild cognitive impairment	 <br>27-30<br><br>18-26	52/74	 <br>29%<br><br>58%	25 (20, 27)

Moderate/severe cognitive impairment	0-17		13%	

*Two patients with missing dynapenia values were able to be recorded as not having sarcopenia given their SMI was recorded as not mypoenic. ADL, activities of daily living; CCI, Charlson Comorbidity Index; GA, geriatric assessment; IADL, instrumental activities of daily living; IQR, interquartile range; MoCA, Montreal Cognitive Assessment; PG-SGA, Patient-Generated Subjective Global Assessment; SMI, skeletal muscle index; SPPB, Short Physical Performance Battery; TUG, Timed Up and Go; WLGS, weight loss grading system; 6-MWT, 6-minute walk test.

Table S2. Baseline characteristics of the study cohort (N=78)

Characteristic	No.	%	Median (Min-max)
**Age at diagnosis**	**78**		73 years (60-93)
**Sex**	**78**		
Male	45	58%	
Female	33	42%	
**Race/Ethnicity**	**78**		
White	62	79%	
Black	10	13%	
Hispanic	3	4%	
Asian	3	4%	
**AML disease category**	**78**		
De novo	53	68%	
Therapy-related AML	12	15%	
AML progressed from MDS or MPN	13	17%	
**ELN 2024 less-intensive risk**	**78**		
Favorable	44	56%	
Intermediate	21	27%	
Adverse	13	17%	
**Molecular Testing for myeloid mutations by NGS**	**78**		
TP53	15	19%	
IDH 1/2	16	21%	
NPM1	14	18%	
FLT3-ITD	10	13%	
NRAS	9	12%	
KRAS	4	5%	
**Laboratory Analysis at Enrollment**	**78**		
WBCx10^3/uL			4.5 (0.4-200.8)
ANCx10^3/uL			1.0 (0-6.1)
Hemoglobin (g/dL)			7.9 (4.5-12.3)
Platelet count			36,000 (2,000-456,000)
Creatinine (mg/dL)			1.0 (0.5-7.8)
Uric acid (md/dL)			4.5 (0.9-17.2)
Lactate Dehydrogenase (U/L) [normal <270]			306 (4.6-488)

Ferritin (ng/mL)			836.5 (46-15,901)
CRP (mg/L) [normal <5]			15 (3-404)
**ECOG PS**	**78**		
0	17	22%	
1	36	46%	
2	9	12%	
3	11	14%	
4	5	6%	
**Treatment Regimen**	**78**		
**Less-intensive regimens**	**64**	81%	
Azacitidine+venetoclax	52	66%	
Decitabine+venetoclax	1	1%	
Azacitidine + Venetoclax+ Enasidenib	1	1%	
Azacitidine + Venetoclax + Gilteritinib	3	4%	
Azacitidine + Venetoclax + Revumenib	2	2%	
Azacitidine + Venetoclax + Ruxolitinib	1	1%	
Revumenib	1	1%	
Gilteritinib	3	4%	
**Intensive regimens**	**7**	9%	
7+3	4	5%	
7+3+Quizartinib	1	1%	
7+3+Gemtuzumab ozogamicin	1	1%	
7+3+Midostaurin	1	1%	
**Deceased before able to initiate induction**	7	9%	
**Allogeneic Stem Cell Transplant in CR1**	15	19%	

AML, acute myeloid leukemia; ANC, absolute neutrophil count; CR, complete remission; ECOG PS, Eastern Cooperative Oncology Group performance status; ELN, European LeukemiaNet; MDS, myelodysplastic syndrome; MPN, myeloproliferative neoplasm

Table S3. Missing data from baseline and follow up sarcopenia measure and geriatric assessment.

GA Domain	Missing at Diagnosis	Reason for Missing at diagnosis	Missing at end of C1	Reason for Missing at end of C1
**Sarcopenia, cachexia, and nutrition**				
**Myopenia**	0	N/A	35	**Death prior to repeat assessment:** 19 (54%)<br>**Transfer of care (transferred/transitioned locally):** 7 (20%)<br>**Patient too busy:** 5 (14%)<br>**Readmitted locally:** 2 (6%)

				**Study team error:** 1 (3%)<br>**Fear of radiation:** 1 (3%)
**Dynapenia**	6	**Patient refused:** 1 (17%)<br>**Error by study team:** 1 (17%)<br>**Decompensated and unable to perform:** 4 (66%)	41	**Death prior to repeat assessment:** 19 (46%)<br>**Transfer of care (transferred/transitioned locally):** 8 (20%)<br>**Patient too busy:** 9 (22%)<br>**Intubated/critically ill:** 1 (2%)<br>**Readmitted locally:** 2 (5%)<br>**Study team error:** 2 (5%)
**Sarcopenia**	4*	**Missing dynapenia measure due to:** decompensated and unable to perform (2) refused (1), and study team error (1).	39	**Death prior to repeat assessment:** 19 (49.0%)<br>**Transfer of care (transferred/transitioned locally):** 7 (18%)<br>**Patient too busy:** 9 (26.0%)<br>**Readmitted locally:** 2 (5.0%)<br>**Study team error:** 1 (3%)<br>**Fear of radiation:** 1 (3%)
**WLGS**	0	N/A	28	**Died prior to repeat assessment**: 19 (68%)<br>**Transfer of care (transferred/transitioned locally)**: 5 (18%)<br>**Patient too busy**: 1 (4%)<br>**Readmitted locally**: 2 (7%)<br>**Study team error**: 1 (4%)
**PG-SGA**	5	**Decompensated and unable to fill out questionnaire**: 5 (100%)	38	**Died prior to repeat assessment**: 19 (50%)<br>**Patient too busy**: 6 (16%)<br>**Transferred care locally/closer to home**: 4 (11%)<br>**Transitioned follow-up care locally**: 4 (11%)<br>**Readmitted locally**: 2 (5%)<br>**Study team error**: 2 (5%)<br>**Intubated**: 1 (2%)
**Labs**	0	N/A		
		**Physical function**		
**6-MWT**	11	**Patient on airborne precautions/confined to their room:** 6 (55%)<br>**Presented with calf abscess:** 1 (9%)	37	**Death prior to repeat assessment:** 19 (51%)<br>**Transfer of care (transferred/transitioned locally):** 7 (19%)<br>**Patient too busy:** 5 (14%)<br>**Patient refused** 2 (5%)

		**Physical therapist felt it was unsafe:** 1 (9%)<br>**Patient refused:** 2 (18%)<br>**Error by study team:** 1 (9%)		**Intubated/critically ill:** 1 (2%)<br>**Readmitted locally:** 2 (5%)<br>**Study team error:** 1 (2%)
**SPPB**	2	**Presented with calf abscess:** 1 (50%)<br>**Error by study team:** 1 (50%)	37	**Death prior to repeat assessment:** 19 (51%)<br>**Transfer of care (transferred/transitioned locally):** 7 (19%)<br>**Patient too busy:** 5 (14%)<br>**Patient refused** 2 (5%)<br>**Intubated/critically ill:** 1 (2%)<br>**Readmitted locally:** 2 (5%)<br>**Study team error:** 1 (2%)
**TUG**	4	**Presented with calf abscess:** 1 (25%)<br>**Patient refused:** 2 (50%)<br>**Error by study team:** 1 (25%)	36	**Death prior to repeat assessment:** 19 (53%)<br>**Transfer of care (transferred/transitioned locally):** 6 (17%)<br>**Patient too busy:** 5 (14%)<br>**Patient refused** 2 (6%)<br>**Intubated/critically ill:** 1 (3%)<br>**Readmitted locally:** 2 (6%)<br>**Study team error:** 1 (3%)
		**Functional Status**		
**Katz ADL/Lawton IADL**	4	**Patient decompensated and unable to fill out questionnaire:** 4 (100%)		
		**Comorbidities**		
**CCI**	0	N/A		
		**Cognition**		
**MOCA**	4	**Patient decompensated and unable to fill out questionnaire**: 4 (100%)		

*Sarcopenia was scored even without the dynapenia value if the patient was not myopenic.

Table S4. Differences in treatment outcomes by sarcopenia, myopenia, dynapenia, and impairment in physical function

Outcome	60-day mortality		90-day mortality		Ability to finish 2 cycles of HMA/Ven, n=67*		CR/CRi, n (%)		Receipt of AlloSCT in CR1		Length of Stay, mean (SD) days		Discharged to rehab, n (%)		Readmission in 100 days, n (%)	
**Whole cohort (n=78)**	20 (25.6%)		24 (30.8%)		40 (60%)		38 (49%)		15 (19%)		18.8 days (10.6)		4 (5%)		37 (47%)	
**Sarcopenia**																
Present (n=22)	10 (45%)	**0.01**	12 (55%)	**0.01**	6 (35%)	**0.01**	5 (23%)	**0.01**	0 (0%)	**0.01**	19.8 (8.7)	0.76	2 (13%)	0.27	12 (54%)	0.15
Absent (n=52)	7 (14%)		9 (17%)		34 (74%)		33 (63%)		15 (29%)		19.0 (11.5)		2 (4%)		25 (48%)	
**Myopenia**																
Present (n=46)	18 (39%)	**0.01**	20 (44%)	**0.01**	18 (47%)	**0.01**	16 (35%)	**0.01**	5 (11%)	**0.03**	16.7 (9.0)	**0.04**	2 (6%)	1.0	20 (61%)	0.75
Absent (n=32)	2 (6%)		4 (13%)		23 (79%)		22 (69%)		10 (31%)		21.8 (12.1)		2 (7%)		17 (57%)	
**Dynapenia**																
Present (n=36)	12 (33%)	**0.01**	15 (42%)	**0.01**	16 (53%)	**0.01**	14 (39%)	**0.01**	2 (6%)	**0.03**	21 (9.9)	0.28	4 (14%)	**0.04**	19 (68%)	0.23
Absent (n=36)	2 (6%)		3 (9%)		24 (80%)		24 (69%)		13 (37%)		18.4 (11.2)		0 (0%)		18 (53%)	
**TUG**																
Impaired (n=46)	19 (41%)	**<0.001**	22 (48%)	**<0.001**	16 (41%)	**<0.0001**	17 (37%)	**0.01**	2 (4%)	**<0.0001**	19.7 (10.3)	0.34	4 (13%)	0.16	20 (63%)	0.54
Unimpaired (n=28)	0 (0%)		1 (4%)		22 (92%)		19 (68%)		13 (46%)		17.2 (11.5)		0 (0%)		15 (54%)	
**6MWT**																
Impaired (n=50)	18 (35%)	**0.01**	22 (43%)	**0.01**	23 (51%)	**0.01**	19 (37%)	**0.02**	3 (6%)	**<0.0001**	18.5 (10.4)	0.45	4 (11%)	0.31	24 (63%)	0.31
Unimpaired (n=16)	0 (0%)		0 (0%)		12 (92%)		13 81%)		11 (69%)		16.2 (11.5)		0 (0%)		10 (63%)	
**SPPB**																
Impaired (n=45)	18 (39%)	**0.001**	1 (3%)	**0.01**	19 (48%)	**0.01**	16 (35%)	**0.01**	2 (4%)	**<.0001**	19.2 (10.3)	0.64	4 (13%)	0.11	19 (59%)	0.83
Unimpaired (n=31)	20 (44%)		3 (10%)		21 (84%)		21 (70%)		13 (43%)		18.1 (11.4)		0 (0%)		17 (57%)	

Continuous variables are presented as mean (SD); categorical variables as n (%). P values compare impaired vs non-impaired or present vs absent groups within each domain. AML, acute myeloid leukemia; alloSCT, allogeneic stem cell transplantation; CR, complete remission; CRi, complete remission with incomplete hematologic recovery; HMA/Ven, hypomethylating agent plus venetoclax; SD, standard deviation; TUG, Timed Up and Go; 6MWT, 6-minute walk test; SPPB, Short Physical Performance Battery.
*The outcome is not relevant to, and therefore excludes, the following: subjects intensively treated (7), single agent Gilteritinib (3), single agent Revumenib (1).

Table S5. Response and cause of death for those subjects with early mortality events

Subject number	Days to death (from treatment initiation, or from diagnosis for those unable to initiate treatment)	Treatment	30-day mortality	60-day mortality	90-day mortality	Best Response to first line therapy	Received 2 cycles of HMA+Venetoclax based regimen	Cause of death
3	31	Azacitidine+Venetoclax	No	Yes	Yes	PR	No	RSV PNA
10	2	Azacitidine+Venetoclax	Yes	Yes	Yes	PD	No	PD
11	54	Azacitidine+Venetoclax	No	Yes	Yes	MLSF	No	Gastrointestinal bleed
12	3	Died before treatment initiation	Yes	Yes	Yes	PD	No	PD
16	4	Azacitidine+Venetoclax+Ruxolitinib	Yes	Yes	Yes	SD	No	Volume overload/hypoxic RF
19	13	Azacitidine+Venetoclax	Yes	Yes	Yes	SD	No	ARDS
20	27	Azacitidine+Venetoclax	Yes	Yes	Yes	SD	No	Cardiac arrest
22	20	Hydroxyurea only, did not start AML directed therapy	Yes	Yes	Yes	PD	N/A	PD
25	82	Azacitidine+Venetoclax	No	No	Yes	SD	Yes	PD
27	13	Azacitidine+Venetoclax	Yes	Yes	Yes	SD	No	Hypoxic respiratory failure
34	9	Died before treatment initiation	Yes	Yes	Yes	PD	No	PD
36	5	Azacitidine+Venetoclax	Yes	Yes	Yes	SD	No	Septic shock
40	68	Azacitidine+Venetoclax	No	No	Yes	SD	No	Septic Shock
48	4	Hydroxyurea only, did not start AML directed therapy	Yes	Yes	Yes	PD	N/A	PD/Leukostasis
53	22	Revumenib	Yes	Yes	Yes	SD	N/A	PD
61	17	Gilteritinib	Yes	Yes	Yes	SD	N/A	Septic shock
68	8	Died before being able to start Ivosidenib	Yes	Yes	Yes	PD	N/A	PD
69	10	Azacitidine+Venetoclax	Yes	Yes	Yes	SD	No	PD
70	6	Gilteritinib	Yes	Yes	Yes	SD	N/A	Hypoxic Respiratory failure

71	11	Azacitidine +Venetoclax+Gilteritinib	Yes	Yes	Yes	SD	No	Hypoxic respiratory failure
72	4	Died before treatment initiation	Yes	Yes	Yes	PD	N/A	PD/leukostasis
73	12	Died before treatment initiation	Yes	Yes	Yes	PD	N/A	Intracerebral hemorrhage
74	66	Azacitidine +Venetoclax	No	No	Yes	SD	No	PD
75	74	Azacitidine +Venetoclax+Gilteritinib	N0	No	Yes	SD	Yes	Hypoxic Respiratory failure

PD, progressive disease; SD, stable disease; MLFS, morphologic leukemia free state.

Table S6. Univariate and multivariable models evaluating sarcopenia, myopenia, dynapenia, and physical function and their impact on treatment outcomes among subgroups

Outcome	60-day mortality: Hazard Ratio [95% CI], *p*-value	90-day mortality: Hazard Ratio [95% CI], *p*-value	Ability to finish 2 cycles of HMA/Ven: Odds Ratio [95% CI], *p*-value	CR/CRi: Odds Ratio [95% CI], *p*-value	Receipt of AlloSCT: Odds Ratio [95% CI], *p*-value	Overall survival: Hazard Ratio [95% CI], *p*-value
**Sarcopenia***						
Univariate	3.50 [1.30, 9.43], **p=0.01**	3.56 [1.47, 8.62], **p=0.01**	0.19 [0.06 0.64], **p=0.01**	0.17 [0.05, 0.53], **p=0.01**	**Inestimable, 0 events in sarcopenic group**	2.03 [1.13, 3.64], **p=0.02**
Whole cohort	1.96 [0.52, 7.42], p=0.32	2.53 [0.79, 8.13], p=0.12	0.19 [0.04, 0.95], **p=0.04**	0.24 [0.07, 0.89], **p=0.03**	**Inestimable, 0 events in sarcopenic group**	1.63 [0.71, 3.73]. p=0.21
Subgroup: ECOG PS 0-2	1.79 [0.30, 10.57], p=0.52	4.26 [0.85, 21.40] p=0.07	0.26 [0.05, 1.47], p=0.13	0.29 [0.06, 1.34], p=0.11	**Inestimable, 0 events in sarcopenic group**	1.17 [0.47, 2.91], p=0.74
Subgroup: Received AML-directed therapy	2.24 [0.50, 10.05], p=0.29	3.02 [0.83, 10.93], p=0.09	0.19 [0.04, 0.94], **p=0.042**	0.17 [0.04, 0.72], **p=0.016**	**Inestimable, 0 events in sarcopenic group**	1.66 [0.76,3.61], p=0.21
Subgroup: Received less intensive therapy	2.29 [0.52, 10.03], p=0.27	2.95 [0.83, 10.43], p=0.09	0.19 [0.04, 0.94], **p=0.042**	0.17 [0.04, 0.73] **p=0.017**	**Inestimable, 0 events in sarcopenic group**	1.25 [0.59, 2.65], p=0.57
**Myopenia**						
Univariate	7.14 [1.65, 31.25], **p=0.01**	4.20 [1.43, 12.35], **p=0.01**	0.24 [0.08, 0.71] **p=0.01**	0.24 [0.09, 0.64], **p=0.01**	0.27 [0.08, 0.88], **p=0.03**	2.00 [1.13, 3.53], **p=0.02**
Whole cohort	5.56 [1.01, 30.48], **p=0.04**	3.99 [1.12, 14.29], **p=0.03**	0.20 [0.04, 0.95], **p=0.04**	0.25 [0.07, 0.92], **p=0.04**	0.30 [0.07, 1.42], **p=0.13**	1.57 [0.82,3.02], p=0.18
Subgroup: ECOG PS 0-2	3.09 [0.43, 22.34], p=0.26	6.12 [0.85, 43.78], p=0.07	0.29 [0.06, 1.51], p=0.14	0.44 [0.12, 1.64], p=0.22	0.29 [0.05, 1.56], p=0.15	1.46 [0.77, 2.77], p=0.24
Subgroup: Received AML-directed therapy	4.05 [0.72,22.75], p=0.11	3.44 [0.91, 12.99], p=0.07	0.21 [0.04, 1.04], p=0.06	0.28 [0.08, 0.99], **p=0.04**	0.30 [0.07, 1.42], p=0.13	1.67 [0.92, 3.03], p=0.09
Subgroup: Received less intensive therapy	4.25 [0.79, 22.80], p=0.09	3.55 [0.97, 12.97], p=0.06	0.21 [0.04, 1.04], p=0.06	0.23 [0.07,0.81], **p=0.02**	0.14 [0.02, 0.95], **p=0.04**	1.57 [0.80, 3.09], p=0.19
**Dynapenia***						
Univariate	6.37 [1.41, 28.57], **p=0.02**	5.65 [1.62, 19.61], **p=0.01**	0.29 [0.09, 0.90], **p=0.03**	0.29 [0.11, 0.78], **p=0.01**	0.10 [0.02, 0.48], **p=0.01**	1.62 [0.90, 2.89], p=0.11
Whole cohort	3.14 [0.59, 16.67], p=0.18	2.00 [0.51, 7.87], p=0.32	0.82 [0.20, 3.32], p=0.78	0.57 [0.14, 2.26], p=0.42	0.178 [0.02, 1.41], p=0.10	0.94 [0.42, 2.12], p=0.89
Subgroup: ECOG PS 0-2	2.70 [0.44, 16.67], p=0.29	5.13 [0.96, 27.78], p=0.06	0.55 [0.110, 2.693], p=0.46	0.64 [0.13, 3.12], p=0.58	0.148 [0.01 1.68], p=0.12	1.00 [0.42, 2.39], p=0.99
Subgroup: Received AML-directed therapy	2.98 [0.55, 16.13], p=0.21	3.18 [0.80,12.66], p=0.10	0.82 [0.20, 3.32], p=0.78	0.57 [0.14, 2.26], p=0.42	0.18 [0.02, 1.41], p=0.10	0.88 [0.40, 1.93], p=0.75
Subgroup: Received less intensive therapy	2.96 [0.33, 26.80], p=0.33	3.99 [0.60, 26.33], p=0.15	0.49 [0.09, 2.79], p=0.42	0.44 [0.11, 1.77], p=0.25	0.29 [0.04, 2.10], p=0.22	0.86 [0.40, 1.85], p=0.69
**TUG**						
Univariate	**Inestimable, 0 events in unimpaired group**	17.31 [2.33,28.9], **p=0.01**	0.063 [0.01, 0.31], **p<0.001**	0.28 [0.10, 0.75], **p=0.01**	0.05 [0.01,0.26], **p<0.001**	2.65 [1.44, 4.87], **p=0.01**
Whole cohort	**Inestimable, 0 events in unimpaired group**	15.26 [1.69, 138.12], **p=0.02**	0.18 [0.03, 1.10], p=0.06	0.86 [0.18, 4.08], p=0.11	0.09 [0.01, 0.74], **p=0.02**	2.28 [1.05, 4.95]. **p=0.04**
Subgroup: ECOG PS 0-2	**Inestimable, 0 events in unimpaired group**	19.18 [1.97, 186.82], **p=0.01**	0.16 [0.03, 1.02], **p=0.05**	0.81 [0.17, 3.92], p=0.08	0.04 [0.01, 0.65], **p=0.02**	2.34 [1.07, 5.11]. **p=0.03**
Subgroup: Received AML-directed therapy	**Inestimable, 0 events in unimpaired group**	18.7 [1.91, 183.30], **p=0.01**	0.18 [0.03, 1.10], p=0.06	0.86 [0.18, 4.08], p=0.11	0.09 [0.01, 0.74], **p=0.02**	2.18 [1.00 4.80], **p=0.05**
Subgroup: Received less intensive therapy	**Inestimable, 0 events in unimpaired group**	10.02 [1.16, 86.34], **p=0.04**	0.14 [0.02, 0.88], **p=0.04**	0.77 [0.15, 3.82] , p=0.75	0.80 [0.01, 1.37], p=0.08	1.84 {0.82, 4.13}, p=0.14
**6MWT**						
Univariate	**Inestimable, no events for non-impaired group**	**Inestimable, no events for non-impaired group**	0.09 [0.01, 0.73], **p=0.02**	0.14 [0.4, 0.54], **p=0.01**	0.03 [0.01, 0.14], **p<.0001**	2.61 [1.21, 5.61], **p=0.014**
Whole cohort	**Inestimable, no events for non-impaired group**	**Inestimable, no events for non-impaired group**	0.26 [0.02, 3.33], p=0.30	0.26 [0.031, 2.22], p=0.22	0.002 [<0.001, 0.37], **p=0.02**	1.73 [0.63, 4.78], p=0.29
Subgroup: ECOG PS 0-2	**Inestimable, no events for non-impaired group**	**Inestimable, no events for non-impaired group**	0.23 [0.02, 2.92], p=0.26	0.24 [0.03, 2.12], p=0.20	0.006 [<0.001, 0.698], **p=0.035**	1.75 [0.62, 5.00], p=0.29
Subgroup: Received AML-directed therapy	**Inestimable, no events for non-impaired group**	**Inestimable, no events for non-impaired group**	0.26 [0.02, 3.33], p=0.30	0.26 [0.03, 2.22], p=0.22	0.002 [<0.001, 0.374], **p=0.02**	1.786 [0.637, 5.004], p=0.27
Subgroup: Received less intensive therapy	**Inestimable, no events for non-impaired group**	**Inestimable, no events for non-impaired group**	0.23 [0.02, 3.02], p=0.26	0.25 [0.03, 2.28], p=0.22	0.005 [<0.001, 0.56], **p=0.03**	1.59 [0.58, 4.36], p=0.36
**SPPB**						
Univariate	13.82 [1.84, 103.86], ***p*=0.01**, c=0.70	5.36 [1.58, 18.13], **p=0.01**	0.17 [0.050, 0.59], **p=0.01**	0.23 [0.09, 0.61], **p=0.01**	0.06 [0.01, 0.29], **p=<0.001**	2.66 [1.45, 4.88], **p=0.01**

Whole cohort	5.29 [0.60, 46.71], p=0.13	2.02 [0.51, 8.00], p=0.32	0.57 [0.13, 2.43], p=0.44	0.83 [0.21, 3.29], p=0.79	0.21 [0.03, 1.43], p=0.11	1.57 [0.75, 3.28]. p=0.23
Subgroup: ECOG PS 0-2	4.09 [0.43, 39.18], p=0.22	1.50 [0.33, 6.74], p=0.60	0.45 [0.10, 2.51], p=0.71	1.32 [0.28, 6.24], p=0.73	0.26 [0.03, 2.04], p=0.20	1.53 [0.69, 3.41]. p=0.295
Subgroup: Received AML-directed therapy	5.18 [0.57, 47.50], p=0.15	1.79 [0.44, 7.38], p=0.42	0.57 [0.13, 2.43], p=0.44	0.83 [0.21, 3.29], p=0.79	0.21 [0.03, 1.43], p=0.11	1.43 [0.68, 3.03]. p=0.35
Subgroup: Received less intensive therapy	3.40 [0.40, 29.32], p=0.26	1.52 [0.39, 5.89], p=0.54	0.46 [0.10, 2.11], p=0.32	0.78 [0.18, 3.28], p=0.73	0.24 [0.03, 1.85], p=0.17	1.21 [0.57, 2.56], p=0.62

The multivariable model for the impact of sarcopenia on 60-day mortality was prespecified to adjust for age, ELN 2024 risk category, ECOG PS, treatment intensity (intensive or less-intensive regimen), and GA variables with prior literature supporting their impact on treatment tolerance (MOCA, CCI, IADL, and SPPB). We also applied these models to the other treatment tolerance outcomes, 90-day mortality and ability to receive 2 cycles of HMA+venetoclax within 90 days. Similarly, we applied these covariates to myopenia and dynapenia given they are components of sarcopenia. Final models for the impact of sarcopenia, myopenia, and dynapenia on response, receipt of alloSCT, and OS and all models for physical function tests included: age, performance status, European LeukemiaNet 2024 less-intensive risk classification, and treatment intensity. Additional covariates, including CCI, MOCA, IADL, and SPPB, were tested but not retained in the final model due to instability. AML, acute myeloid leukemia; alloSCT, allogeneic stem cell transplantation; CR, complete remission; CRi, complete remission with incomplete hematologic recovery; ECOG PS, Eastern Cooperative Oncology Group performance status; HMA/Ven, hypomethylating agent plus venetoclax; SD, standard deviation; TUG, Timed Up and Go; 6MWT, 6-minute walk test; SPPB, Short Physical Performance Battery.

Table S7. Sensitivity analysis models for sarcopenia using a stepwise model building procedure

Outcome	Covariates Included in Final Model (with Sarcopenia)	Sarcopenia point estimate, [95% CI], p-value, c-statistic, EPV ratio
60-day Mortality	ELN 2024 Risk Category; SPPB	3.06 [0.83, 11.29], p=0.09, c=0.81, EPV=6.7
90-day Mortality	ELN 2024 Risk Category; MoCA; ADL	4.67 [1.54, 14.17], p=0.01, c=0.82, EPV=6.0
Ability to Complete 2 Cycles of HMA/Venetoclax	ELN 2024 Risk Category; CCI; SPPB	0.18 [0.05, 0.72], p=0.04, c=0.80, EPV=10
Receipt of Allogeneic Stem Cell Transplantation (AlloSCT)	ELN 2024 Risk Category; SPPB	**Inestimable, 0 events in sarcopenic group**
Complete Remission/Complete Remission with Incomplete Hematologic Recovery (CR/CRi)	ELN 2024 Risk Category; CCI; ADL	0.15 [0.04,0.64], p=0.01, c=0.89, EPV=9.5
Overall Survival (OS)	ELN 2024 Risk Category; Treatment Intensity; ADL	1.47 [0.78, 2.77], p=0.24, c=0.76, EPV=13.8

Results of a sensitivity analysis using a stepwise model-building procedure, evaluating the following candidate variables (sarcopenia, ELN 2024 risk category, ECOG performance status, treatment intensity, MOCA, Charlson comorbidity index, ADLs, and SPPB). ELN 2024 Disease Risk Score was prespecified to be included in the models. A p-value of 0.25 was used to determine the variables retained in the final model while maintaining an acceptable c-statistic larger than 0.7. EPV, event-per-variable ratio.

Table S8. Sensitivity analysis using myopenia as a continuous variable

Outcome	60-day mortality: Hazard Ratio [95% CI], *p*-value	90-day mortality: Hazard Ratio [95% CI], *p*-value	Ability to finish 2 cycles of HMA/Ven: Odds Ratio [95% CI], *p*-value	CR/CRi: Odds Ratio [95% CI], *p*-value	Receipt of AlloSCT: Odds Ratio [95% CI], *p*-value	Overall survival: Hazard Ratio [95% CI], *p*-value
**Myopenia**						
Univariate	0.96 [0.91, 1.01], p=0.15, c=0.60	0.96 [0.92, 1.01], p=0.11, c=0.61	1.08 [1.01, 1.16], p=0.03, c=0.65	1.08 [1.02, 1.15], p=0.01, c=0.66	1.10 [1.02, 1.18], p=0.02, c=0.67	0.97 [0.94, 1.001], p=0.059, c=0.59
Multivariable	0.99 [0.92,1.07], p=0.86, c=0.78	0.97 [0.90,1.04], p=0.37, c=0.74	1.08 [1.01, 1.18], p=0.05, c=0.82	1.08 [0.99, 1.17], p=0.09, c=0.88	1.22 [1.05,1.42], p=0.01, c=0.96	0.99 [0.95, 1.02], p=0.43, c=0.76

Table S9. Association with myeloid gene mutations with presence of sarcopenia and myopenia.

**Sarcopenia**	**Present (n, %)**	**Not present (n, %)**	**p-value**
TP53	2 (9%)	8 (15%)	0.71**
NPM1	5 (23%)	9 (17%)	0.75**
RAS	4 (18%)	9 (17%)	1.00**
ASXL1	5 (23%)	10 (19%)	0.76**

IDH1	3 (14%)	11 (21%)	0.54**
FLT3	5 (23%)	9 (17%)	0.75**
RUNX1	3 (14%)	15 (29%)	0.16*
Splicing Mutations	7 (32%)	17 (32%)	0.94*
**Myopenia**	**Present**	**Not present**	**p-value**
TP53	9 (20%)	4 (13%)	0.41*
NPM1	9 (20%)	5 (15%)	0.66*
RAS	9 (20%)	5 (15%)	0.66*
ASXL1	10 (22%)	6 (19%)	0.75*
IDH1	8 (1%)	7 (22%)	0.62*
FLT3	7 (15%)	7 (22%)	0.45*
RUNX1	6 (13%)	12 (38%)	**0.012***
Splicing Mutations	12 (26%)	14 (44%)	0.10*

*Chi-square test, **Fisher's exact test

Table S10. ELN Risk 2024 association with sarcopenia and myopenia

**Sarcopenia**	**Present**	**Not present**	**p-value**
Favorable	11 (50%)	33 (64%)	0.21*
Intermediate	9 (41%)	11 (21%)	
Adverse	2 (9%)	8 (15%)	
**Myopenia**	**Present**	**Not present**	**p-value**
Favorable	24 (52%)	20 (63%)	0.61*
Intermediate	13 (28%)	8 (25%)	
Adverse	9 (20%)	4 (13%)	

*Chi-square test,
**Fisher's exact test

Table S11. ELN Risk 2022 association with sarcopenia and myopenia.

**Sarcopenia**	**Present**	**Not present**	**p-value**
Favorable	3 (14%)	5 (10%)	0.48*

Intermediate	7 (32%)	11 (21%)	
Adverse	12 (55%)	36 (69%)	
**Myopenia**	**Present**	**Not present**	**p-value**
Favorable	5 (11%)	3 (9%)	1.00**
Intermediate	11 (24%)	7 (22%)	
Adverse	30 (65%)	22 (69%)	

*Chi-square test,
**Fisher's exact test

Table S12. Pearson's correlation coefficients for myopenia, dynapenia, malnutrition, cachexia, inflammation, age, and physical function metrics.

**Variable**	**Muscle mass**	**Muscle strength**
	**r^2, p-value**	
PG-SGA	-0.21, *p*=0.08	**-0.45, *p*<0.0001**
WLGS	**-0.24, *p*=0.03**	-0.01, *p*=0.94
6-Minute Walk	**0.23, *p*=0.05**	**0.65, *p*<0.0001**
TUG	-0.17, *p*=0.19	**-0.45, *p*<0.0001**
SPPB	**0.34, *p*=0.01**	**0.63, *p*<0.0001**
Age	-0.20, *p*=0.08	**-0.32, *p*=0.01**
C-reactive protein	0.01, *p*=0.91	**-0.27, *p*=0.03**
Ferritin	-0.05, *p*=0.67	-0.17, *p*=0.16
Muscle mass	N/A	**0.31, *p*=0.01**
Muscle strength	0.18, *p*=0.13	N/A

Higher scores in the PG-SGA and WLGS describe increase in severity of malnutrition and cachexia, respectively. Higher scores in TUG describe worse physical function while lower scores on SPPB and 6-minute walk describe worse physical function. PG-SGA, Patient-generated Subjective Global Assessment; TUG, Timed Up and Go; 6MWT, 6-minute walk test; SPPB, Short Physical Performance Battery; WLGS, Weight Loss Grading Scale.

Table S13. Change in myopenia, dynapenia, weight, malnutrition, and physical function after 1 cycle of AML treatment

**Measure**	**Median (IQR)**	**Better**	**Same**	**Worse**
Muscle mass (n=43)	-1.93 cm^2/m^2 (-5.3, +0.8)	14 (33%)	0 (0%)	29 (67%)
Muscle strength	-1.7 kg (−2.0, +7.0)	12 (32%)	2 (6%)	23 (62%)
Weight (n=51)	−1.6 kg (−5.0, +0.3)	15 (29%)	3 (6%)	33 (65%)
WLGS (n=50)	0 points (−1, +1)	16 (32%)	17 (34%)	17 (34%)
PG-SGA (n=40)	+0.5 points (−3, +6)	15 (37%)	5 (13%)	20 (50%)
TUG (n=31)	+0.3 seconds (-2.8, +1.8)	15/31 (48%)	0 (0%)	16/31 (52%)
SPPB (n=39)	-0.8 points (-2.0, +1.0)	13 (33%)	9 (23%)	17 (44%)
6MWT (n=31)	-17.1 ft (-109, +104)	19 (61%)	5 (16%)	7 (23%)

An increase in TUG time is a worse performance. Clinically meaningful changes for SPPB (+/- 1 point) and 6MWT (+/-50 feet) are established from prior literature. For the rest of the variables, any change was used to define better or worse. IQR, Interquartile range; TUG, Timed Up and Go; 6MWT, 6-minute walk test; SPPB, Short Physical Performance Battery.

Figure S1. Consort diagram for NCT05458258

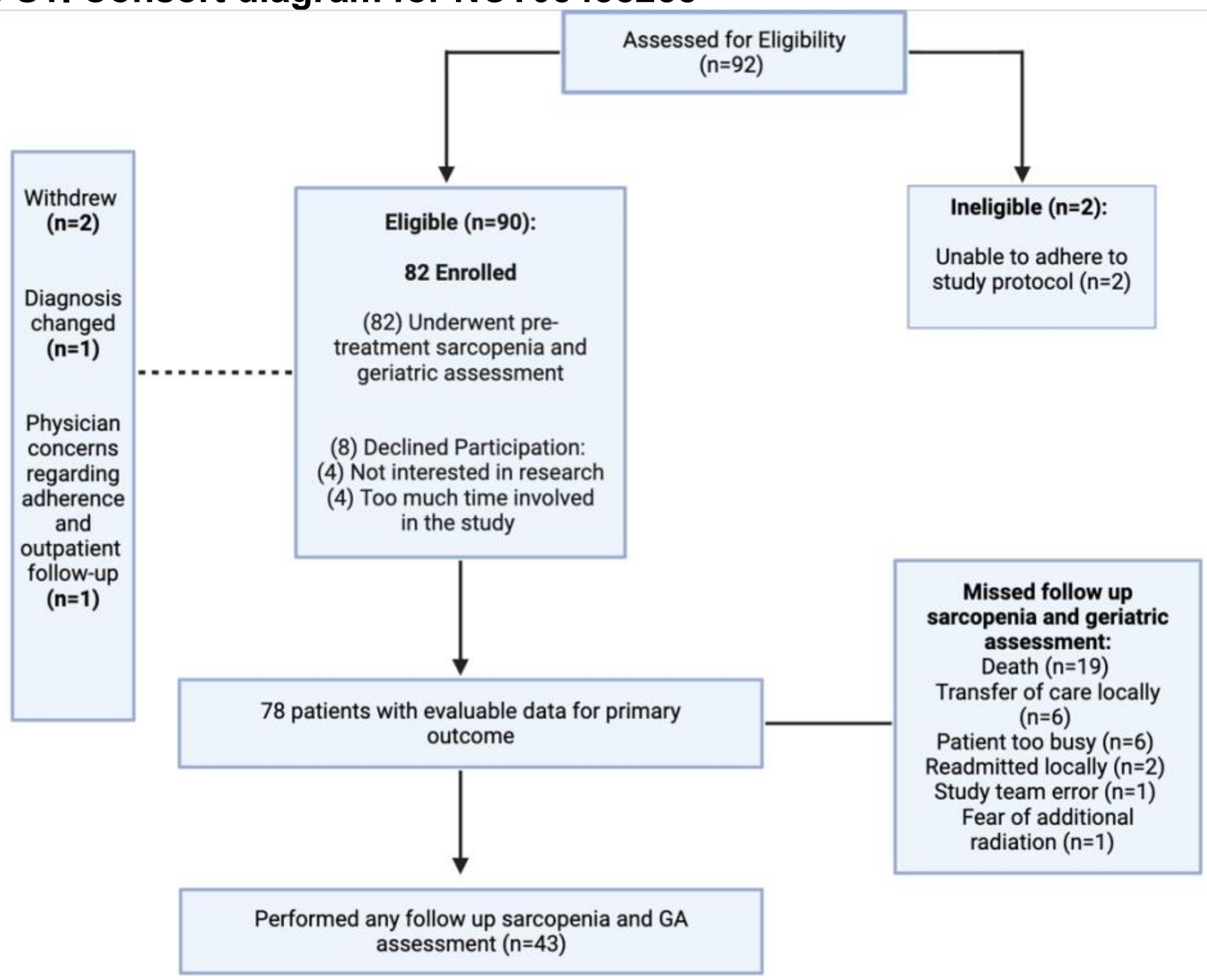

Figure S2. Overall survival stratified by physical function measured by the Timed Up and Go test. Those impaired on the TUG test had shorter survival in the whole cohort and pre-specified subgroups. The sample size for each curve is: whole cohort (n=73), ECOG Performance Status 0-2 (n=57), received AML-directed therapy (n=66), received Less-intensive

treatment regimen (n=59).

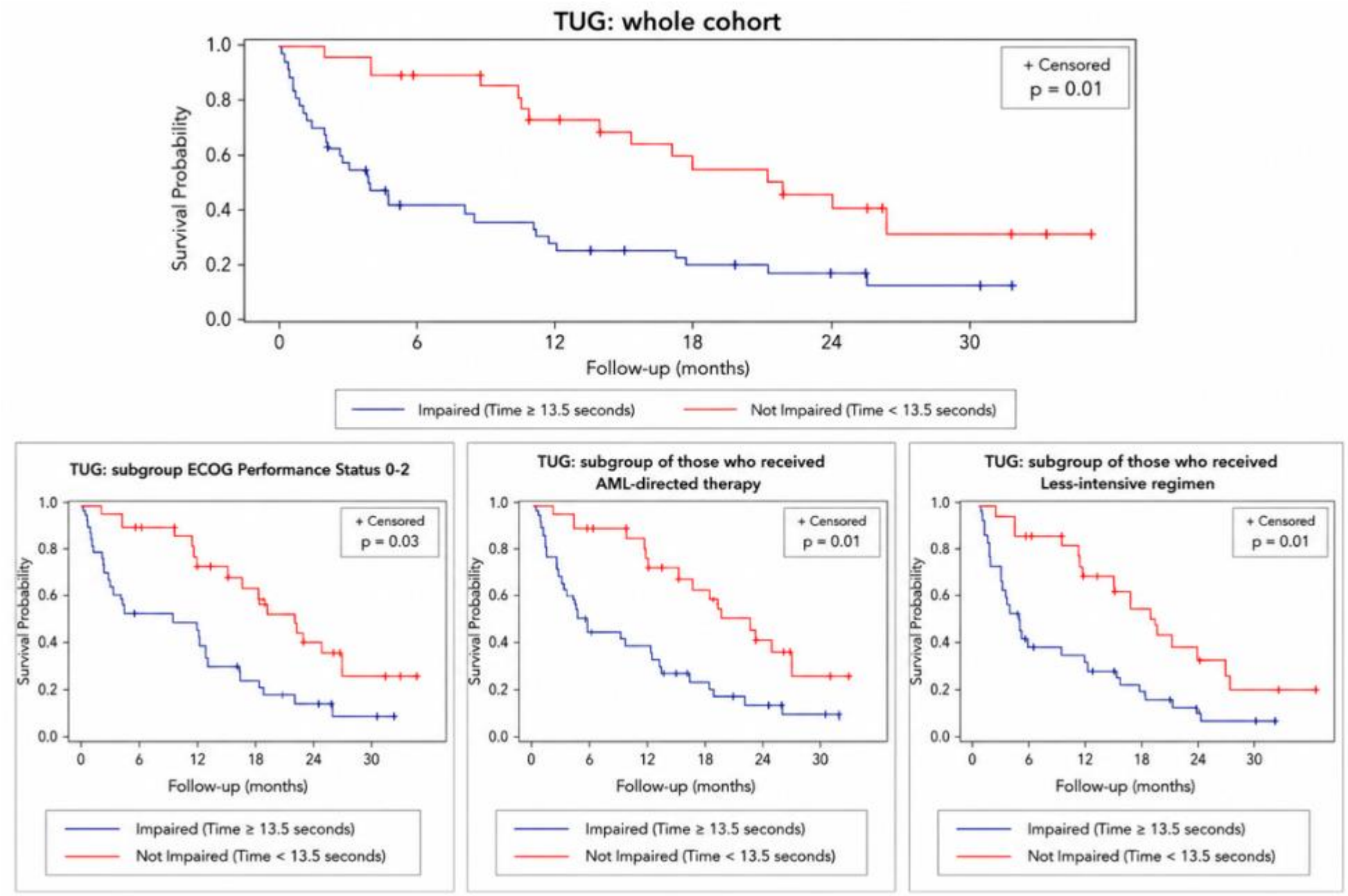

Supplement References

1. Cruz-Jentoft AJ, Bahat G, Bauer J, Boirie Y, Bruyère O, Cederholm T, et al. Sarcopenia: revised European consensus on definition and diagnosis. Age Ageing. 2019;48(1):16-31.
2. Martin L, Senesse P, Gioulbasanis I, Antoun S, Bozzetti F, Deans C, et al. Diagnostic Criteria for the Classification of Cancer-Associated Weight Loss. Journal of Clinical Oncology. 2014;33(1):90-9.
3. Jager-Wittenaar H, Ottery FD. Assessing nutritional status in cancer: role of the Patient-Generated Subjective Global Assessment. Current opinion in clinical nutrition and metabolic care. 2017;20(5):322-9.
4. Enright PL, McBurnie MA, Bittner V, Tracy RP, McNamara R, Arnold A, et al. The 6-min walk test: a quick measure of functional status in elderly adults. Chest. 2003;123(2):387-98.
5. Guralnik JM, Simonsick EM, Ferrucci L, Glynn RJ, Berkman LF, Blazer DG, et al. A short physical performance battery assessing lower extremity function: association with self-reported disability and prediction of mortality and nursing home admission. Journal of gerontology. 1994;49(2):M85-94.
6. Podsiadlo D, Richardson S. The timed "Up & Go": a test of basic functional mobility for frail elderly persons. J Am Geriatr Soc. 1991;39(2):142-8.
7. Katz S. Assessing self-maintenance: activities of daily living, mobility, and instrumental activities of daily living. J Am Geriatr Soc. 1983;31(12):721-7.
8. Lawton MP, Brody EM. Assessment of older people: self-maintaining and instrumental activities of daily living. Gerontologist. 1969;9(3):179-86.
9. Charlson M, Szatrowski TP, Peterson J, Gold J. Validation of a combined comorbidity index. J Clin Epidemiol. 1994;47(11):1245-51.
10. Nasreddine ZS, Phillips NA, Bédirian V, Charbonneau S, Whitehead V, Collin I, et al. The Montreal Cognitive Assessment, MoCA: a brief screening tool for mild cognitive impairment. J Am Geriatr Soc. 2005;53(4):695-9.
